# A longitudinal investigation of dietary diversity during the COVID-19 pandemic in Mandinka households in Kanifing, Brikama, and the West Kiang region in The Gambia

**DOI:** 10.3389/fnut.2022.907969

**Published:** 2022-09-30

**Authors:** Richard Sidebottom, Solomon Bizuayehu Wassie, Carla Cerami, Momodou W. Jallow, Shailaja Fennell, Sarah Dalzell

**Affiliations:** ^1^Department of Land Economy, University of Cambridge, Cambridge, United Kingdom; ^2^Department of Agricultural Economics, Bahir Dar University, Bahir Dar, Ethiopia; ^3^Nutrition Unit, MRC Gambia at London School of Hygiene and Tropical Medicine, Banjul, Gambia; ^4^MRC Nutrition and Bone Health Research Group, University of Cambridge, Cambridge, United Kingdom

**Keywords:** nutrition, COVID-19, dietary diversity, food security, coping mechanisms

## Abstract

The Covid pandemic has exposed fissures of inequality through heightened food insecurity and nutritional deficiency for vulnerable social cohorts with limited coping mechanisms. Given the multi-dimensional pathways through which its effects have been felt, several researchers have highlighted the need to analyse the pandemic in specific contexts. Using random and fixed effect regression models, this study analyzed longitudinal survey data collected from 103 Mandinka households in rural and urban Gambia. The study employed convenience and snowball sampling and involved the monthly collection of detailed income, food consumption, expenditure, sourcing, migration, health, and coping mechanism data through mobile phone interviews which yielded 676 observations. Food insecurity was manifest in terms of quality, not quantity, and spread unevenly across food types and households. Dietary outcomes and sourcing strategies were associated with location, improved sanitation, household size, changes in monthly income, Covid policy stringency, and Covid cases but these associations varied by food group. Staples were the most frequently consumed food group, and dark green vegetables were the least. Rural communities were more likely to eat more healthy millets but much less likely to consume dairy products or roots and tubers. Access to own production was also important for Vitamin A-rich foods but higher incomes and markets were key for protein and heme-iron-rich foods. Tighter Covid policy stringency was negatively associated with dietary diversity and, along with fear of market hoarding, was positively associated with reliance on a range of consumption and production coping mechanisms. Resilience was higher in larger households and those with improved water and sanitation. The number of Covid cases was associated with higher consumption of protein-rich foods and greater reliance on own produced iron-rich foods. Very few households received Government aid and those that did already had access to other income sources. Our findings suggest that the nature of food insecurity may have evolved over time during the pandemic. They also reiterate not only the importance of access to markets and employment but also that the capacity to absorb affordability shocks and maintain food choices through switching between sources for specific nutritious food groups varied by household and location.

## Introduction

The impacts of the COVID-19 pandemic have been routed via *direct* pathways to physical and mental well-being and *indirect* ones through reduced employment, income, and consumption (1–4). Their magnitude, nature, and duration have exposed pre-existing fissures of inter and intra-country economic and health inequality most vividly manifest in heightened food insecurity (5–9). The pandemic has highlighted the need for policy makers to focus on the links between nutritional diversity and health (10, 11) and livelihood resilience during external shocks (12).

From the outset of the pandemic, many countries witnessed changes in food supply and demand. Early impacts were often contingent upon state support, food markets, social networks, and nutritional knowledge (9). Thereafter, food insecurity evolved along with country-specific pathways (13). Several studies have highlighted a preference for online food sourcing and home cooking in developed countries (14). The pandemic has also been associated with healthier diets in Mexico (15) but not in Italy (16) or the United Kingdom (5, 17) in Ethiopia, the immediate effects of policy restrictions appear to have been short-lived (18), but elsewhere in Sub-Saharan Africa (SSA) lockdown restrictions may have exacerbated food insecurity through income shocks, poor targeting of social welfare, and the undermining of long-term resilience (19).

In many countries, income and food effects persisted long after policy easing (20). Subsequent pandemic phases saw a shift to less expensive foods with shorter supply chains (13) as many countries reported restrictions on specific food choices but not overall availability (14). Irrespective of policy regime or food system disruption, the impacts of the pandemic have been unevenly distributed across numerous divides. Dou et al. (14) have highlighted varying resilience capacity *within* social and income cohorts, not simply across them. In Bangladesh, income and diet deterioration were most evident amongst rural residents, informal workers, and the less well-educated (21). Further research highlighted the importance of age, occupation, and gender, rather than location, as these are associated with pre-existing food insecurity (22).

One driver of such resilience is access to alternative food sources. There is voluminous literature on the relative attributes of production, income, or market pathways to dietary diversity under normal circumstances (23). However, spatial and temporal fluidity suggest that people switch between complementary sources depending on food type, season, and the nature of the external shocks (24). In India and SSA, pandemic coping mechanisms included the use of agriculture as an income and food source substitute (25, 26). In Burkina Faso, Nigeria, and Ethiopia, Madzorera et al. (27) found that crop production was associated with stable dietary diversity in the pandemic, whilst non-producers' diets were more vulnerable to affordability shocks.

The incomplete separation between urban and rural spaces and livelihoods ties in with the idea “migration-food security nexus” (28, np), which allows for fluidity of people, occupations, money, and food as part of food security coping strategies. The potency of the pandemic's multi-dimensional employment, income, and expenditure impact pathways (14) is therefore likely to be linked with household-specific livelihood and sourcing strategies, not just food environments or lockdown regimes. To appreciate the dynamic and intricate nature of these interactions requires an understanding of specific contexts (20).

## Method

### Study background

The Migration, Nutrition and COVID-19 (MNC19) study sought to contribute to this understanding through a longitudinal investigation of the indirect impacts of the pandemic in The Gambia from November 2020 to September 2021. Impacts were assessed in terms of perceived threat, coping strategies, and nutritional outcomes. The study was an interdisciplinary collaboration as part of the Research on Millets and Nutritional Enhancement Traits for Iron bioavailability project (MillNET_i)^1^. Design, training, and coordination were led by researchers from the Department of Land Economy at the University of Cambridge and the MRC Nutrition and Bone Health Research Group in the United Kingdom, and the survey was conducted by members of the Nutrition Theme in the MRC Gambia at London School of Hygiene and Tropical Medicine, The Gambia. Data analysis was completed through co-operation between the Universities of Cambridge and Bahir Dar, Ethiopia.

The Gambia's socio-economic geography makes it an informative case study. Ranked 174th in terms of the Human Development Index (HDI), The Gambia is a relatively urbanized service-driven economy reliant upon tourism and remittances (29). The country's predominantly Muslim population of 2.3 million is made up of several large ethnic groups – Mandinka, Fula, Wolof, and Jola (30). With significant levels of domestic and international mobility, there is a high dependence upon remittances of food and money but foreign cashflows were expected to fall by 20% during the pandemic (31, 32). This was expected to exacerbate a pre-existing triple burden of malnutrition that was manifest in the form of dietary insufficiency, excess, and low quality. This has contributed to a high incidence of diabetes, hypertension, and iron deficiency (33, 34). With a semi-arid climate and low agricultural capacity, the Gambia imports 40% of its cereals, especially rice (34). In terms of health, reliance on transport, employment, incomes, and diets, the country was therefore vulnerable to an external shock (32, 35–38).

**Figure 1:**
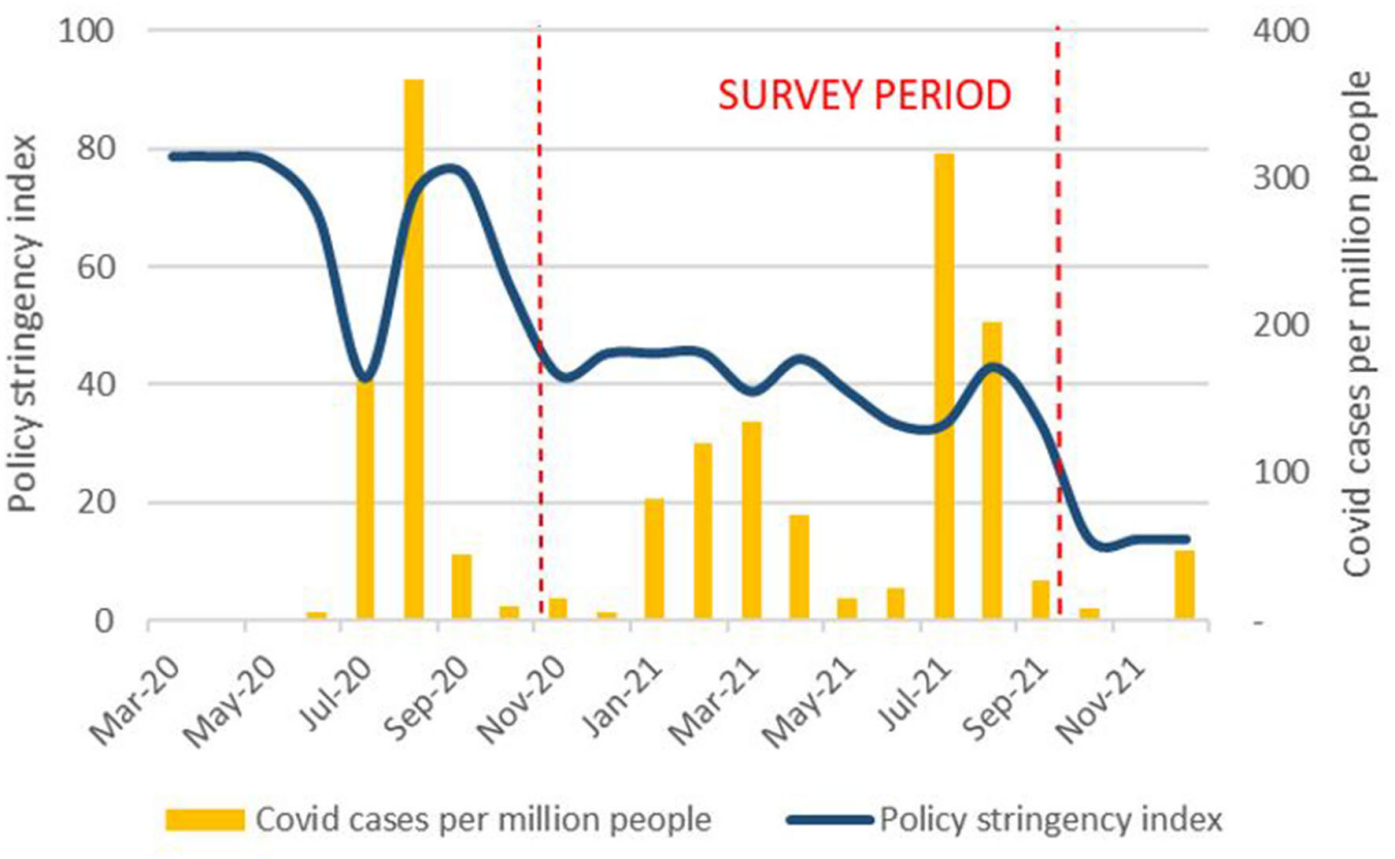
Policy stringency and Covid cases [Own graph, data sources (40, 41)].

The Gambia saw a spike in reported COVID-19 cases from July to September 2020, a smaller rise from January to April 2021 and another spike after June 2021 (39). During the period of the MNC19 study, there was 260% increase in cumulative cases. By 30 September 2021, there had been 9,935 cases and 338 fatalities (40). However, according to the Oxford COVID-19 Government Response Stringency Index^2^, Gambian restrictions were never as severe as those in India or parts of Europe and were relaxed after September 2020, despite a rising number of Covid cases (41) (Figure 1).

In early 2020, the national incidence of food insecurity rose from 5% in 2016 (33, 34) to 25% (6). By July 2020, this fell to 20% (42), in part due to the provision of government food aid. The easing of policy restrictions allowed children to return to school but subsequent phases of the pandemic have seen the continued erosion of incomes through job losses, enforced job switching, or lower remittances. Seasonal factors temporarily eased employment concerns but the rural poor remained three times more likely to be food insecure due to low affordability (43).

### Study design

Our research objective was to unpack the drivers of these pockets of food insecurity as the pandemic unfolded in terms of perceived threats, coping strategies, and nutritional outcomes. To realize our objective, monthly household food consumption, income, expenditure, and migration data were collected from November 2020 to September 2021. Data collection began *after* initial Gambian lockdown restrictions began to ease but *before* case numbers started to accelerate. With a high dependence upon remittances, imported food, and foreign tourism, we hypothesized that the effects of the pandemic were as likely to come via indirect pathways (both perceived and actual) and direct ones. Given the initial spread of the pandemic was concentrated outside The Gambia, indirect effects were likely to *precede* direct ones.

### Sampling, data collection, and ethics

Our unit of analysis was the household which we defined as those who regularly shared cooking facilities in accordance with Gambian government surveys (44, 45)^3^. Our design sought to reflect the “multi-nodal” nature of these households, which requires researchers to rethink the notion of location and context (28). We, therefore, surveyed both urban and rural households and those that we had reason to believe contained members who were geographically mobile. We recruited Mandinka households through randomized selection from an urban convenience sample previously used by one of the authors^4^ in Brikama and Kanifing. This was supplemented by adopting a snowballing technique from urban contacts to identify rural respondents in the West Kiang District of Central River Region. Snowballing within one ethnic group enabled us to identify intra-group interlinkages and control for inter-group dietary variations. As the survey was conducted remotely, we were restricted to those who had mobile phone access and whose numbers were still in use.

Prior to data collection, households were informed they would be called approximately once a month for up to 12 months. They were advised that there was no reward for participation or penalty for non-participation or non-completion. Respondents were given 24 h to consider their participation before the survey commenced. If they agreed^5^, audio-recorded informed consent was obtained from both the household head and any other nominated adult respondent who would complete all or part of the survey. A total of 106 households were recruited but three dropped out (due to sickness or migration) after the initial call, leaving 60 urban households and 43 rural. We collected data^6^ on household composition, income, migration and expenditure, coping strategies and consumption, and the sourcing of 35 food items chosen and categorized in accordance with FAO guidelines (46) (Table 1). Recruitment and questionnaires were completed remotely by mobile phone throughout the entire study. Data were collected on tablets using Redcap survey software (47). Enumerator training was conducted remotely by lead researchers and in person by local supervisors under appropriate social distancing protocols. Training involved seminars, discussions, and pilot interviews which provided feedback on the list of foods and question content and ordering. All procedures were approved by the Scientific and Ethical committees of MRC Gambia/London School of Hygiene and Tropical Medicine, the Gambia Government/MRC Joint Ethics Committee, and by the Department of Land Economy at the University of Cambridge.

**Table 1:** Food list for consumption questions.

	**Food group**		**Food item**
1	*Staples*	1	Rice (Mano): mono, nyakatango, fajiringo, benechin, other rice
		2	Millets (Sanyo/Suno)
		3	Fonio (Findo)
		4	Maize (tubanyo): cob, roasted, futo, nyelengo
		5	Sorghum (kinto): nyelengo, futo
		6	Bread
		7	Pasta
2	*Roots & Tubers*	8	White roots and tubers
3	*Nuts, Pulses, legumes*	9	Groundnuts
		10	Pulses
		11	Nuts and seeds
4	*Dairy*	12	Milk and other dairy products
5	*Eggs*	13	Eggs: from Chicken, duck, guinea fowl or other
6	*Fish*	14	White fish
		15	Bony fish
		16	Canned fish
		17	Shellfish: Oyster (Nganya), mussels, sea snail, crabs, shrimps, lobster
7	*Meat*	18	Flesh meat
		19	Canned meat
		20	Organ meat: liver, kidney, heart and/or other organ meats
8	*Vegetables*	21	Orange Veg and Tubers rich in Vitamin A: Carrot, Red pepper, Pumpkin, Orange Sweet potatoes, orange vegetables
		22	Dark green leafy vegetables: Baobab leaf (naa/lalo), sorrel (kucha/domoda), amaranth (morongo), spinach, water leaf, cassava leaf, okra (kanjo), Moringa (nebedayo) and/or other dark green leaves
		23	Other vegetables
9	*Fruit*	24	Orange fruits rich in Vitamin A
		25	Other Fruits
10	*Sweets*	26	Tea/coffee with sugar
		27	Sugary drinks
		28	Cakes, biscuits/cookies, pastries
		29	Other sweets
11	*Oils & Fats*	30	Groundnut oil
		31	Palm oil
		32	Palm kernel oil
		33	Vegetable oil
		34	Margarine/butter
12	*Condiments/Spices*	35	Condiments/Spices

## Data analysis

### Data cleaning

Due to a range of pandemic-related problems and the nature of the snowballing process, recruitment lasted from mid-November, 2020 until the start of March 2021. We started recording income and consumption data immediately and not all households could be reached at every round or each call made in a particular calendar month. To address these unbalanced panel data, we applied two sets of filters. As there were only 26 recruits prior to 31 December 2020, we excluded income and consumption data before then but included household background information. For all others, we excluded calls less than 20 days apart. Of 779 surveys completed, this left us with 676 observations across 103 households. We had at least five observations for 97 households but with some time gaps. Of the six with only three or four valid observations, four were uncontactable after May and the other two were infrequently available (Table 2).

**Table 2:** Phone call schedule.

**Data collection period**	**Households (HH) recruited**					**Total calls**	**Households called**
	**Start**	**New**	**Drop outs with data**	**Drop-outs no data**	**Net**		
Nov–Dec 2020	0	26		1	25	0	0
Jan-21	25	17		2	40	43	38
Feb-21	40	47			87	85	73
Mar-21	87	16	1		102	110	93
Apr-21	102		2		100	90	90
May-21	100		1		99	97	91
Jun-21	99		10		89	89	84
Jul-21	89		12		77	52	52
Aug-21	77		16		61	45	42
Sep-21	61		0		61	65	61
		106		3	103	676	103
**Phone call frequency distribution**
**Number of calls per household**		**Percentage of sample**	**Cumulative**
3 or 4	6	5.8%	6%
5	13	12.6%	18%
6	26	25.2%	44%
7	37	35.9%	80%
8	14	13.6%	93%
9	7	6.8%	100%

### Variable specification

#### Dependent variables

We used a range of nutritional proxies to test the robustness of our findings (Table 3). The *Household Food Insecurity Access Scale (HFIAS)* assesses a household's psychological experience of food access over the previous 4 weeks (48). Although open to response bias (49), questions on *perceived* food insecurity form a useful complement to other indices measuring *actual* consumption behavior within a given socio-cultural context. Respondents were asked how frequently they worried about food availability; a number of questions about dietary diversity (including whether they had not been able to eat preferred foods, forced to eat non-preferred foods, or eat a more limited variety); and questions regarding the impact of having insufficient food (50). In terms of actual consumption over the previous 7 days, we calculated *Food Consumption (FCS)*^7^ and *Food Consumption Nutrition* scores *(FCS-N)*. FCS categorizes food item frequencies into eight groups weighted in accordance with their calorific and nutritional content (50). FCS-N provides a more direct indication of Vitamin A, protein, and heme-iron (He) intake (51). We also used our food sourcing data to calculate FCS and FCS-N scores by market and own production.

**Table 3:** Variable specifications.

**Data type**	**Description**	**Specification**	**Variable name**
**Control**	Location	Urban yes /no	Location
	Gender household head	Male Yes/no	HHHgender (male)
	Age household head	Numeric	HHHAge
	Education household head	None yes/no	Educdummy
	Health household head	Self-reported diabetes or hypertension	HHHhealthstart
	Improved water supply	Yes/no	HHImpwaterdummy
	Improved sanitation	Yes/no	HHImptoiletdummy
**Independent**	Household size	Total number of residents	Hhsize
	Household dependency ratio	Ratio non-working age residents to working age residents	Depratio
	Resident health change	Self-reported any new health conditions for any resident	Anyresidentsick
	Resident migration	Absolute migration in and out	Mobility
	Cash expenditure	Fish money per resident (Dalasi)	Fishmoney (GMD)
	Income change	Income up yes/no	Income up
	Income source: employment	Cited as a top 3 income source	Employment
	Income source: business	Cited as a top 3 income source	Business
	Income source: remittances	Cited as a top 3 income source	Remittance
	Covid policy measures	Oxford Policy stringency index monthly data	Policystring
	Covid cases	Monthly National cases per million John Hopkins data	covidcases
	Covid perceived impact	Hoarding cited yes/no	Hoarding
**Dependent**	Household Food Insecurity Access Scale	Each of nine questions scored (0–3) depending on the frequency of response (Never, rarely, sometimes, often). Sum is HFIAS score (0–27)	HFIAS
	Food Consumption score (FCS) all sources	Sum of Staples (2); Pulses (3); Veg (1); Fruit (1); Meat/Fish (4); Milk (4); Sugar (0.5); Oil (0.5) all sources. Max 7 each group; weights in brackets	FCS^1^
	FCS market sources	As FCS, market sources only	FCSmarket
	FCS own production sources	As FCS, own production only	FCSown
	FCS-Nutrition (FCS-N) Protein all sources	Pulses; Milk and dairy; organ meat; flesh meat; fish; and eggs	Protein
	FCS-N Protein Market sources	As FCS-protein, market sources	Proteinmkt
	FCS-N Protein^2^ own production	As FCS-protein, own production	Proteinown
	FCS-N Vitamin A^3^ all sources	Milk, dairy; Organ meat; eggs; Orange vegetables; dark green leafy vegetables; Vitamin A rich orange fruits	VitA
	FCS-N Vitamin A Market sources	As FCS-VitA, market sources	VitAmkt
	FCS-N Vitamin A own production	As FCS-VitA, own production	VitAown
	FCS-N Heme^4^ iron all sources	Flesh meat and fish	Iron
	FCS-N Heme iron Market sources	As FCS-iron, market sources	Ironmkt
	FCS-N Heme iron own production	As FCS-iron, own production	Ironown

1 Food Consumption Score is calculated for foods sourced from own production and the market, as well as the total.

2 Food consumption Nutrition Score for Protein is calculated for foods sourced from own production and the market, as well as the total.

3 Food consumption Nutrition Score for vitamin A is calculated for foods sourced from own production and the market, as well as the total.

4 Food consumption Nutrition Score for Heme iron is calculated for foods sourced from own production and the market, as well as the total.

GMD, is the official currency of the Republic of Gambia; HFIAS, Household Food Insecurity Access Scale; FCS, Food Consumption Score; FCS-N, Food Consumption Nutrition Score; FCS-Protein, Food Consumption Nutrition score for Protein-rich foods; FCS-VitA, Food Consumption Nutrition score for Vitamin A rich foods; FCS-Iron, Food Consumption Nutrition score for Heme-iron rich foods.

#### Independent variables

We defined an income trend variable which had a value of 1 if income had risen compared to the previous month, or 0 otherwise. Each of employment, business, and remittance income was assigned a value of 1 if it was cited as a top 3 source in the previous month, or 0 otherwise^8^. For expenditure, we adopted the Gambian concept of “fish money” that was a commonly used indicator of a household's monthly disposable cash for spending on food. This was normalized by dividing by the number of household members in each round. We used the absolute sum of in- and out-migration to gauge population fluidity and changes in household dependency ratios. Perceived and actual external risks were measured using the number of times food hoarding was cited as perceived impact^9^, the Oxford COVID-19 Government Response Stringency Index (41), and the official number of national Covid cases per million (40). We also used a number of control variables – household head characteristics (gender, age, education); location (rural or urban); and services (improved water and sanitation). For health, we allowed for pre-existing self-reported health conditions of the household head and the incidence of changed health conditions for any household member each month. We also included a specific control variable for observations during Ramadan (12 April to 12 May).

### Econometric model specification

We acknowledge issues of endogeneity and causality. According to Holland (52), *x* is said to have an effect on *y* if the following three conditions are met (i) *y* follows *x* temporally, (ii) y changes as *x* changes (relationship is statistically significant), and (iii) no other causes should eliminate the relation between *x* and *y*, referred in the literature as an omitted variable (53). While the first two conditions are arguably accounted for in the study, we did not roll out the third condition associated with model specification. To this end, we have included location dummies and months in the regression to account for location and time-specific factors that are not observable and referred to in the literature as unobserved heterogeneity. However, we acknowledge that there may be other factors that may, for example, affect covid cases and outcome variables simultaneously.

Panel data allow control for unobservable intra-household factors or variables that change over time but not inter-household heterogeneity. The most commonly used techniques to analyse panel data are fixed effects and random effects models (54). A fixed effects model assumes that household level factors may influence the outcome variable and hence need to be controlled. Once the effect of time-invariant characteristics has been accounted for to avoid omitted variable bias, we can assess the net effect of the predictors on the outcome variable. A fixed effects model also assumes that the time-invariant features are unique to a household and should not be correlated with other household characteristics. More formally, a fixed effects model can be specified as:


(1) $$\begin{array}{l} Y_{it}={\text{β}}_{1}X_{it}+\alpha_{i}+u_{it} \end{array}$$

where *Y*_*it*_ the dependent variable; *X* represents explanatory variables; α_*i*_ (*I* = 1, 2, 3, … *n*) is unknown household-specific intercept and n is the number of households; β are coefficients to be estimated; *i* indexes households and *t* indexes time; and *u*_*it*_ is the error term. Unlike the fixed effects model, a random effects model assumes that the variation across entities is random and uncorrelated with the explanatory variables. The advantage of a random effects model is that you have a chance to include time-invariant or household level variables in the regression (53). However, this may lead to omitted variable bias.

Formally, the model can be specified as:


(2) $$\begin{array}{l} Y_{it}=\text{β}X_{it}+\alpha +\xi_{it}+e_{it} \end{array}$$

where ξ_*it*_ is between household error term, *e*_*it*_ is within household error term, and others as defined above.

The estimation procedure depends on the outcome variable. We used multiple linear regression for continuous outcome variables and negative binomial models for count outcome variables. Although Poisson regression models could be used for the latter, the negative binomial model does not restrict the variance to be equal to the mean. This is referred to as “overdispersion” and measured by “alpha” in the estimation model. If ‘alpha' is significant, the negative binomial is preferred.

Our selection of random or fixed effects models was guided by a Hausman specification test, which adopts a null hypothesis that the household level error term (ξ_*it*_) is not correlated with the regressors, in which case a random effects model is used (53).

## Results

### Descriptive statistics

#### Household backgrounds

The total number of 1,406 residents meant that the average initial household size was just under 14 (Table 4). Urban households were 40% larger than rural households but dependency ratios were lower; 98% of residents were Mandinka, 2% Fula, and 53% female; 46% were born in the Mandinka region of Kiang West and a further 30% in the Brikama and Kanifking urban districts; 47% were under 16 years old and only 4% over 65; 68% lived in a compound occupied by a single dwelling and most had water piped to the dwelling or compound and private sanitation.

**Table 4:** Descriptive data.

**Household data**	**All**	**Urban**	**Rural**
Location (*n*)	103	60	43
Location (%)	100%	58%	42%
Households with improved water (%)	82.5%	73.3%	95.3%
Households with improved toilet (%)	83.5%	81.7%	76.7%
Initial household size (mean)	13.7	15.7	11.0
Initial dependency ratio (mean)	1.1	0.8	1.6
Household head male^1^ (%)	87%	87%	84%
Household head age (mean)	57.0	59.3	53.8
Household head education none/primary (%)	56%	50%	65%
Household head education secondary or higher (%)	44%	50%	35%
Household head health condition at start (%)	19.4%	23.3%	14.0%

1 Includes three households with both a male and female head.

A total of 85 households had a sole male head, 12 had a sole female head and in three households headship was shared between one male and female^10^. Most heads had no education, especially in rural areas; 97% were married – two-thirds of marriages were polygamous. The self-reported incidence of hypertension or diabetes was especially evident in urban household heads (23.3%)^11^. Of those who specified a sector, most household heads worked in business, service, construction, or farming. Nearly 40% had multiple occupations, but 17% were unpaid (unemployed or housewives).

#### Pandemic awareness

**Figure 2:**
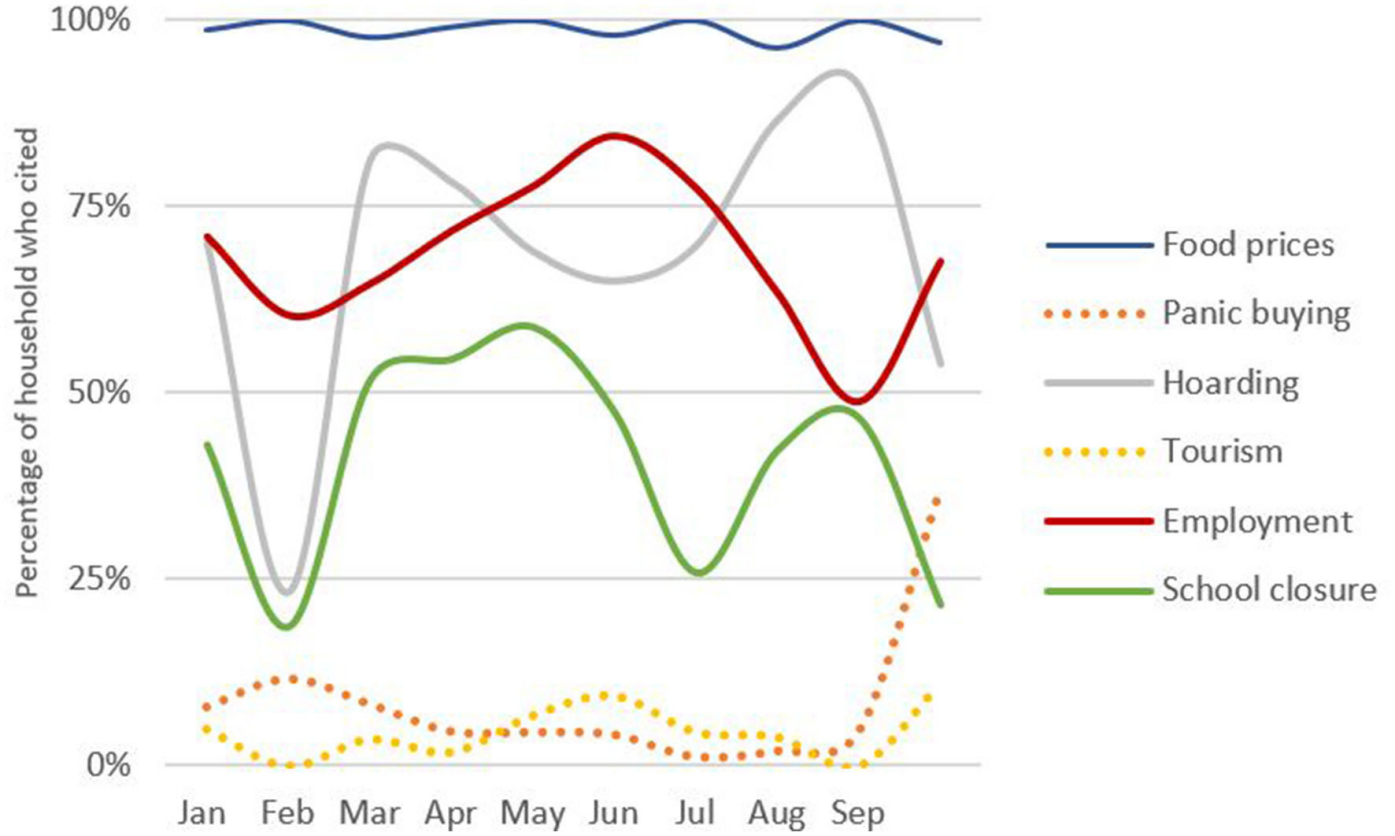
Perceived Covid impacts by month (Own graph, data source Survey data).

To avoid over-attribution of dietary behavior to the pandemic, we were careful not to lead respondents and asked a number of questions regarding perceived household exposure to all types of external shock. 30% of households experienced food shortages during the previous year, usually in July and August. In terms of the current shock, awareness remained high throughout the survey period. Initial fears of the market and school closures and reduced employment eased over time but food prices were a consistent worry and concerns of hoarding rose with the number of Gambian Covid after April 2021 (Figure 2). These patterns reflect the relatively few government restrictions and reliance on food markets (32, 34).

#### Consumption

Our food frequency results were similar to those of the most recent one-off national surveys (33, 34, 42) but also showed variation over time (Table 5). Staples were the most frequently consumed food group, and dark green vegetables were the least. Rice and bread were the most popular staples but more healthy options, such as millets, regularly consumed in Wolof and Fula communities (42) were noted mainly in rural households during harvest periods. Consumption of Vitamin A-rich fruit and vegetables peaked between May and July and was lowest in February and March. Rural consumption of dairy products and roots and tubers was much lower than in urban areas.

**Table 5:** Main Food consumption patterns by location and month.

**Average number of days consumed per week**							
**Food**	**All**	**Urban**	**Rural**	**Jan**	**Feb**	**Mar**	**Apr**	**May**	**Jun**	**Jul**	**Aug**	**Sep**
Rice	6.1	6.8	6.5	6.2	5.9	7.0	6.7	6.9	6.9	4.2	7.0	7.0
Millets	2.0	2.3	2.7	2.9	1.7	2.9	1.9	2.0	3.2	0.8	2.7	2.5
Roots/Tubers	2.9	3.7	1.9	3.7	3.3	3.2	3.2	2.7	2.8	2.1	3.7	2.8
Pulses	1.5	1.7	1.1	1.4	1.4	1.4	1.3	1.5	1.5	1.4	2.3	1.5
Dairy	5.2	5.5	3.7	5.0	3.7	4.2	5.0	5.8	4.9	5.9	5.6	6.1
Eggs	2.5	2.4	1.4	1.8	1.6	1.8	2.0	2.1	2.0	3.9	2.6	3.0
Dark green Vegetables	1.7	1.7	1.5	1.9	1.8	1.8	1.6	1.5	1.6	1.9	1.4	1.6
Vitamin A-rich vegetables	3.6	3.4	1.8	2.8	2.3	2.4	3.3	3.8	2.8	5.7	3.1	3.1
Vitamin A-rich fruit	2.7	3.1	2.7	0.6	0.5	1.2	3.1	5.7	6.3	2.7	2.8	0.7
Other fruit	5.7	6.2	6.2	6.0	6.3	5.9	6.2	6.4	6.0	4.1	6.3	6.4
Flesh meat	1.7	2.3	1.7	1.8	1.3	1.6	2.3	2.7	1.8	1.0	2.6	1.9
Fish	6.2	6.8	6.5	6.9	6.0	7.0	6.8	7.1	7.2	5.1	4.8	6.0
Oils & Fats	5.7	5.7	5.2	5.3	5.2	5.1	6.2	6.2	5.6	6.1	5.3	5.3

All male household heads ate full meals outside the house on average 3 three times a week throughout the survey period. Markets were visited just over 6 days a week and were the main household food source (Table 6). Market reliance was highest in urban areas but visit frequency was lower, especially before the relaxation of policy in March and as Covid cases began to rise after July. Own production was intermittently important for fruit, vegetables, and millets, especially in rural areas and gifting was only noted in urban Vitamin A-rich fruit.

**Table 6:** Main food sourcing patterns by location.

**Food**	**Totals**			**Urban**			**Rural**		
	**Market^1^**	**Own production^2^**	**Gift^3^**	**Market**	**Own production**	**Gift**	**Market**	**Own production**	**Gift**
Rice	97%	3%	0%	99%	1%	0%	92%	8%	0%
Millets	84%	13%	2%	95%	4%	2%	67%	30%	3%
Roots/Tubers	84%	15%	0%	91%	9%	0%	69%	30%	1%
Pulses	83%	15%	2%	90%	8%	2%	71%	26%	3%
Dairy	97%	2%	1%	99%	0%	1%	94%	5%	1%
Eggs	95%	5%	0%	99%	1%	0%	89%	11%	1%
Dark green Vegetables	56%	44%	1%	72%	27%	1%	30%	70%	0%
Vitamin A rich vegetables	91%	9%	0%	96%	4%	0%	83%	17%	0%
Vitamin A rich fruit	38%	56%	6%	45%	50%	5%	26%	68%	6%
Other fruit	86%	13%	2%	89%	10%	1%	77%	19%	5%
Flesh meat	88%	9%	3%	90%	7%	2%	82%	13%	5%
Fish	99%	0%	0%	100%	0%	0%	99%	1%	0%
Oils and fats	100%	0%	0%	100%	0%	0%	100%	0%	0%

1 Refers to food purchased at the market.

2 ^2^Refers to food sources from own production.

3 ^3^Refers to foods sourced from friends or family with no monetary exchange.

We used the data for foods consumed at home over the previous 7 days to calculate a number of food security indices (Table 7). Average *FCS* scores were 12% lower in rural areas (especially in February and March), but average FCS-N rural protein and heme-iron scores were 25% lower and FCS-N Vitamin A scores 29% lower. August saw below-average intakes of Vitamin A and heme-iron-rich foods but not of protein-rich foods. Spatial disparities were most evident in February to March but the widest inter-household dispersion from May to July may also reflect non-locational factors.

**Table 7:** Food consumption indices.

**Month**	**HFIAS**			**FCS**			**FCS-N Protein**			**FCS-N Vit A**			**FCS-N Heme iron**		
	**TOT**	**URB**	**RUR**	**TOT**	**URB**	**RUR**	**TOT**	**URB**	**RUR**	**TOT**	**URB**	**RUR**	**TOT**	**URB**	**RUR**
Jan	1.1	1.1	1.4	88.5	89.8	79.2	23.3	24.2	17.0	12.3	13.0	7.2	11.0	11.3	8.2
Feb	1.4	1.6	1.0	85.2	93.0	72.9	21.2	25.0	15.1	10.2	12.4	6.9	9.4	11.2	6.6
Mar	0.9	1.1	0.8	87.4	94.3	79.4	22.7	27.0	17.8	10.7	13.5	7.5	10.4	12.1	8.3
Apr	0.5	0.4	0.6	94.2	98.5	88.9	23.7	26.5	20.3	14.6	17.6	10.8	10.7	11.7	9.4
May	0.6	0.6	0.6	99.7	101.5	97.4	25.9	27.2	24.1	19.2	19.4	18.8	11.7	12.4	10.9
Jun	0.7	0.8	0.6	95.7	102.3	88.4	24.6	28.5	20.4	17.2	20.0	14.1	10.8	12.4	9.0
Jul	0.3	0.2	0.4	98.7	102.1	93.2	26.1	27.6	23.7	17.5	19.4	14.4	10.7	10.9	10.3
Aug	0.6	0.9	0.1	98.8	104.2	90.6	25.7	28.4	21.8	14.5	17.3	10.4	8.7	9.9	6.9
Sep	0.7	0.7	0.6	93.0	101.3	83.8	24.4	28.4	19.9	13.9	16.2	11.3	9.1	10.5	7.5
**Mean**	0.8	0.8	0.7	93.1	98.1	86.1	24.0	26.9	20.0	14.4	16.4	11.6	10.4	11.6	8.7
**SD**	1.58	1.71	1.36	16.7	13.7	17.9	9.3	8.4	9.1	6.8	6.3	6.4	4.5	4.3	4.3

TOT, Total sample; URB, Urban sample; RUR, Rural sample; HFIAS, Household Food Insecurity Access Scale; FCS, Food Consumption Score; FCS-N Protein, Food Consumption Nutrition score for Protein-rich foods; FCS-N Vit A, Food Consumption Nutrition score for Vitamin A rich foods; FCS-N Heme iron, Food Consumption Nutrition score for Heme-iron rich foods; SD, Standard deviation.

These consumption and sourcing patterns suggest that pandemic impacts may vary by season, location, and food group. This was also evident in our *HFIAS* scores. Our results reveal constraints on food *choice* rather than *quantity*. These were addressed most frequently through the use of savings or borrowing food or money, rather than cutting non-food expenditure or begging. Urban HFIAS scores were higher, especially in February and March but only 3% of observations had a score greater than 4. HFIAS scores were lowest during April (the period of Ramadan) and after July.

However, these scores may reflect a greater ability to borrow cash or food to maintain consumption patterns, not necessarily a greater need.

#### Income and expenditure

Sixty-seven percent of calls witnessed no change in household monthly income and only 14% witnessed declines. February and September saw the highest incidence of falling incomes but the distribution was skewed in favor of net gains in all other months, especially June. However, income falls were concentrated in 47% of households. This was evenly split by location but rural households were nearly twice as likely to experience *multiple* monthly declines and were particularly affected June to September. The most commonly cited reason was usually lower production (irrespective of location) but declining remittances were also significant in July and September.

Employment and business were the most frequently cited income sources^12^ but urban households were twice as likely as rural households to cite business. The importance of remittance receipts (from domestic or international sources) rose after May in urban areas and after July in rural areas. These were usually in monetary form (rather than goods or food) and used to buy food. As such, their receipt may be a key factor in dietary diversity, especially during the lean period and when Covid cases begin to reaccelerate.

However, a diverse range of income sources was not universal. Whilst 74% of households received remittances, 35% of recipients received only once. Government aid was received by <9% of households and accounted for only 2% of observations. Moreover, all households who received aid also received remittances. Income and remittance reliance may also be related to the movement of people; 43% of households had instances of new people entering the household and 57% of people leaving. All 72% of households (especially in urban areas) had some type of resident mobility. This confirms our prior expectation of household fluidity.

**Figure 3:**
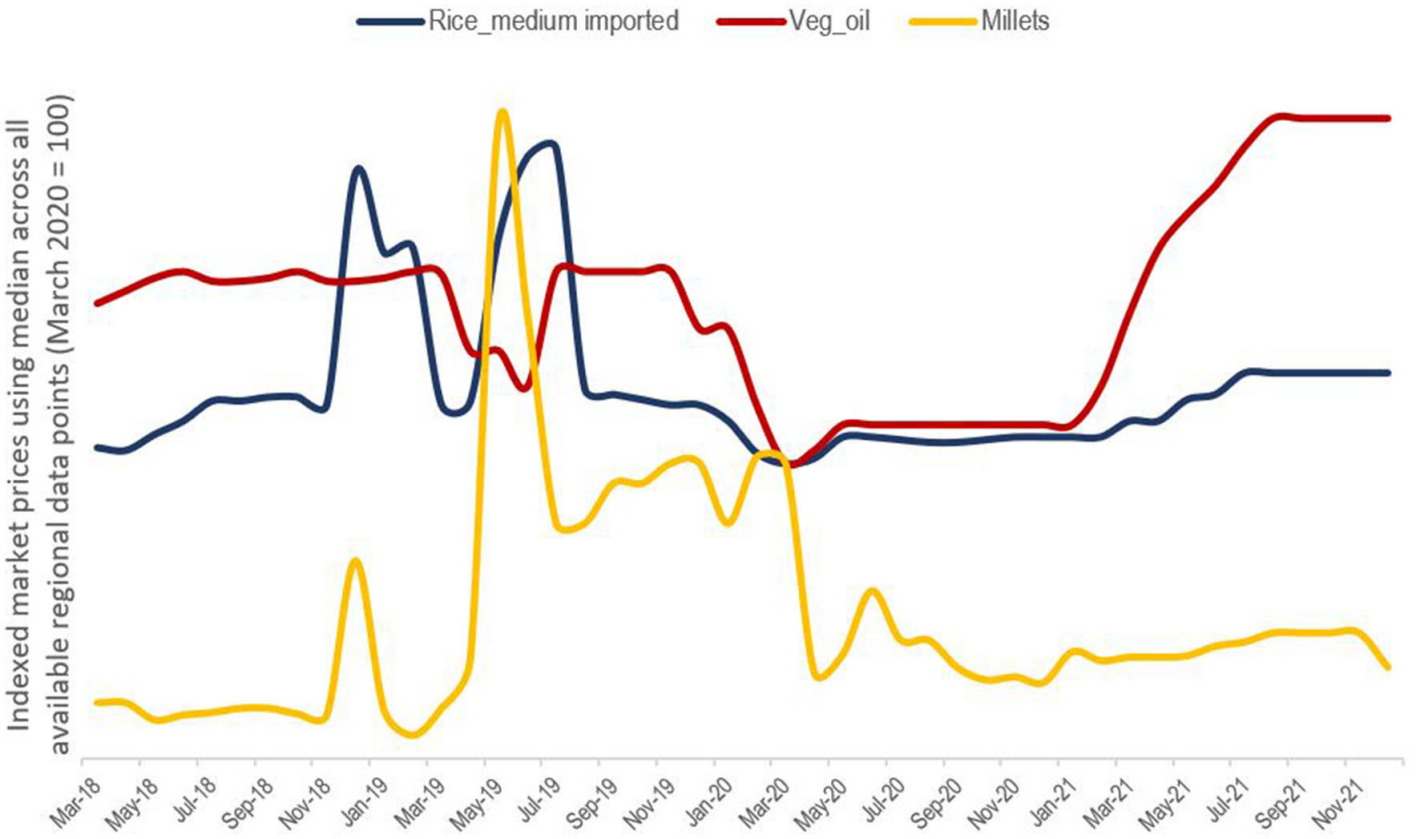
Market food prices [Own graph, date source (55)].

Household budgets were also subject to fluctuations on the expenditure side; 42% of calls recorded increased monthly expenses, especially in August. This was usually attributed to higher rice prices (69%) but also to cooking oil. The affordability of nutritious foods, such as dairy or fish, was seldom mentioned. Based on historical price data (55), the 67% rise in vegetable oil prices since March 2020 appears to be more of a pandemic-specific effect (Figure 3). By contrast, the 14% rise in rice prices is not extraordinary by historical standards and has been offset by the falling cost of millets which to some degree are a staple substitute. However, this did not appear to impact the consumption frequency of either.

The impact of these budgetary fluctuations was gauged by analyzing the Gambian concept of “fish money” – a household's monthly disposable cash. As this was a clearly understood local concept, it was one of the few variables we asked respondents to quantify. As respondents sometimes could not remember or were unwilling to reveal the amount, we collected only 642 estimates but had data for all households. The average of these (normalized for household size) was 449 Gambian Dalasi (GMD) and peaked at 549 GMD in August. However, dispersion around the mean was significant (standard deviation of 391 GMD). The urban average (531) was 1.6 times the rural average (327) and those in the top quintile had more than three times as much fish money as those in the bottom quintile.

Each of our consumption, income, and expenditure indicators, therefore, suggested a degree of spatial, temporal, and inter-household variation.

### Regression analysis

Table 8 summaries the descriptive data for our regression variables, and Table 9 shows our tests of association between 20 independent variables and our 13 dependent variables. They identify a number of significant associations (especially for HFIAS, Vitamin A, Vitamin A market, and Protein market) but the explanatory power is weaker for own production and iron-rich food.

**Table 8:** Variable data for regression model.

**Control variables**			**Independent variables**			**Dependent variables**		
**Name^1^**	**Mean**	**SD**	**Name**	**Mean**	**SD**	**Name**	**Mean**	**SD**
Location^2^	58%	0.5	Anyresidentsick	13%	0.3	HFIAS	0.8	1.6
Household head (male)^3^	88%	0.3	Mobility	14%	0.5	FCS	93.1	16.7
Household head age	57.0	12.0	Fishmoney(GMD)	449	391	FCSmarket	76.0	17.3
Education dummy	53%	0.5	Incomeup	20%	0.4	FCSown	7.3	9.2
Health household head	19%	0.5	Employment	68%	0.5	Protein	24.0	9.3
HHImpwaterdummy	83%	0.3	Business	51%	0.5	Proteinmkt	22.4	9.8
HHImptoiletdummy	83%	0.3	Remittance	33%	0.4	Proteinown	1.4	2.6
Hhsize	13.9	6.6	Policystring	39.6	5.3	VitA	14.4	6.8
Depratio	1.1	0.9	covidcases	110.3	98.9	VitAmkt	11.1	6.4
			Hoarding	70%	0.4	VitAown	3.2	3.8
						Iron	10.4	4.5
						Ironmkt	10.0	4.6
						Ironown	0.3	1.3

1 Refer to Table 3 for variable specifications.

2 58% for location means that 58% of the respondents used in the regression are from urban area (please refer to Table 3 for reference group of dummy variables). Others can also be interpreted with the same fashion.

3 Household head data Includes three households with both a male and female head. SD, standard deviation; GMD, Gambian Dalasi the official currency of the Republic of Gambia; HFIAS, Household Food Insecurity Access Scale; FCS, Food Consumption Score.

**Table 9:** Regression model results.

**Variable**	**HFIAS Scale**	**FCS**			**Vitamin A**			**Protein**			**Heme-Iron**		
		**FCS score**	**Own production sources**	**Market sources**	**FCS-N Vitamin A score**	**Own production sources**	**Market sources**	**FCS-N Protein score**	**Own production sources**	**Market sources**	**FCS-N Heme-iron Score**	**Own production sources**	**Market sources**
Location	0.049	6.421^***^	−5.439^***^	12.548^***^	0.662	−0.947^***^	1.925^***^	0.804^**^	−2.187^***^	1.095^***^	0.162^***^	−0.488	0.186^***^
Gender household head	−0.695^***^	3.879	2.3	1.697	1.106^**^	−0.673^*^	1.119^***^	0.451	0.955	0.154	0.008	−0.019	0.003
Age household head	0.012	0.044	−0.130^**^	0.127	−0.026^*^	−0.001	−0.003	−0.009	−0.048^**^	0.003	0.000	−0.016	0.001
Education household head	0.461^***^	−0.072	−2.558^**^	2.093	−0.156	−0.232	0.234	−0.124	−0.367	0.078	0.005	0.021	0.009
Health household head	0.068	−1.489	−1.013	−1.607	0.17	−0.154	0.052	−0.378	−0.978^***^	−0.166	−0.063	−0.052	−0.071
Improved water supply	0.077	2.328	0.316	2.451	0.933^**^	0.43	1.112^***^	0.713^**^	−0.677	0.461	0.03	0.855^*^	0.011
Improved sanitation	−0.018	4.092^*^	−4.910^***^	7.301^***^	0.287	0.066	1.152^***^	0.444	−1.252^**^	0.671^**^	0.241^***^	0.203	0.219^***^
Household size	0.002	0.355^**^	0.053	0.263^*^	0.051^***^	−0.029	0.030^*^	0.043^***^	0.048	0.031^**^	0.014^***^	0.009	0.015^***^
Household dependency ratio	0.025	−0.918	0.253	−0.716	0.022	0.039	0.014	−0.006	−0.014	0.007	−0.033	−0.209	−0.021
Resident health change	0.485^**^	0.942	0.497	0.148	0.048	−0.121	0.021	−0.028	0.079	−0.048	−0.062	0.575^*^	−0.090^*^
Resident migration	0.004	1.038	0.019	0.431	0.016	0.023	0.063^**^	0.014	0.096	0.021	0.002	0.134	−0.002
Cash expenditure	−0.028	0.249	−0.093	0.233	−0.005	0.019	−0.008	−0.009	−0.102^***^	−0.007	0.002	−0.001	0.004
Income change	−0.225^***^	3.497^***^	0.685	3.032^**^	0.102^**^	−0.024	0.059	0.087^***^	−0.158	0.095^**^	0.091^**^	−0.261	0.106^**^
Income source: employment	−0.746^***^	0.207	0.005	−0.172	−0.004	0.119	0.063	0.006	0.349^*^	−0.011	−0.034	−0.052	−0.041
Income source: business	−0.436^**^	4.141^***^	0.528	1.378	0.092^**^	0.039	0.113^**^	0.077^**^	0.086	0.064^**^	0.014	0.045	−0.007
Income source: remittances	−0.861^***^	3.957^***^	−0.321	1.668	0.036	0.019	0.172^***^	0.117^***^	0.085	0.115^***^	−0.023	−0.008	−0.047
Covid policy measures	0.031^*^	−0.547^***^	−0.247^***^	−0.177	−0.016^*^	−0.075^***^	−0.008^**^	−0.005^*^	−0.018	−0.006^*^	−0.006^*^	−0.061^*^	0.0004
Covid cases	−0.001	−0.007	0.007^**^	−0.020^***^	0.001	−0.001^**^	−0.001^***^	0.0001	0.002^***^	0.0003^**^	0	0.008^***^	−0.001^***^
Covid perceived impact	0.875^***^	−0.018	−0.723	−1.144	−0.008	0.209^*^	−0.032	0.008	−0.096	0.011	−0.012	0.17	−0.004
Ramadan	−0.499^*^	5.036^***^	−0.228	2.943^*^	0.243^***^	0.283^*^	0.125^**^	0.053	0.11	0.045	0.086^**^	0.13	0.054

HFIAS, Household Food Insecurity Access Scale; FCS, Food Consumption Score; FCS-N, Food Consumption Nutrition Score; FCS-Protein, Food Consumption Nutrition score for Protein-rich foods; FCS-VitA, Food Consumption Nutrition score for Vitamin A rich foods; FCS-Iron, Food Consumption Nutrition score for Heme-iron rich foods.

*** Significance at a 99% confidence level.

** Significance at a 95% confidence level.

* Significance at a 90% confidence level.

The most frequently significant independent variables are location, improved sanitation, household size, changes in monthly income, Covid policy stringency, and Covid cases but there was some variation by the dependent variable. Contrary to our *a priori* expectations, we found no association between migration and most dietary metrics which may be attributable to variable specification.

Household Food Insecurity Access Scale was significantly negatively associated with being a male household head (though the number of female heads was small); positive income changes; income from any source; and Ramadan. Although the number of Covid cases had no effect, case numbers only began to rise in August so may not affect all observations. A link between personalized perception of risk and behavior was more apparent in the positive relationship between HFIAS and any resident becoming sick, the fear of hoarding, and the education of the household head.

Food Consumption associations with income changes, Ramadan, policy stringency, business, and remittance income sources mirrored those of HFIAS as expected. Income from employment and hoarding was not significant but we did identify additional positive associations with location, improved sanitation, and household size. However, the direction and magnitude of these associations varied by food source. The negative association between own production sources and location is intuitive but our results also showed a negative relationship with household head age and education, improved sanitation, and Covid policy stringency and a positive one with Covid cases. FCS market was positively associated with location, improved sanitation, household size, positive income changes, and Ramadan but negatively associated with Covid cases.

These relationships may suggest a degree of switching between food sources, as well as a number of household-specific choice constraints, such as age, income, and location. The indirect effects of the pandemic are also implied through the negative association between stringency measures and own production (possibly attributable to restrictions on movement or higher costs of transport back to farms). However, higher Covid cases seem to encourage more reliance on own production. The perceived effects of hoarding do not seem to have a bearing on consumption patterns, only coping mechanisms to maintain them.

Our nine FCS-N nutritional intake variables provided corroboration of our HFIAS and FCS findings that indicated the importance of location, household services (sanitation or water supply), Covid policy stringency, and cases. However, there was some variation by nutrient type. We found a positive association between location and market sourcing for all nutrients and for protein and iron scores but not Vitamin A scores. Covid policy stringency had a negative association with all but two of our FCS-N variables – protein own and iron market^13^.

Covid cases were associated with nutrient sourcing but not nutrient scores. Moreover, the impact was inconsistent. Covid cases had a negative association with Vitamin A rich food consumption from either source and with iron rich foods sourced from the market. This may reflect the seasonal availability of own produced Vitamin A-rich food. By contrast, Covid cases were positively associated with protein scores from both own production and markets (possibly due to people trying to improve health resilience).

We also found a positive association between Vitamin A scores and male household heads, improved water supply, positive income changes, business income, and Ramadan and a negative association with age. Male household heads and larger households with improved water supply were more likely to rely on market sources of Vitamin A, but female-led households those who feared market hoarding were more likely to rely on their own production. However, it should be noted that the number of female-led households was relatively small.

Protein scores were also positively associated with higher monthly income, access to business and remittance income, improved water supply, and household size but not with the gender, age, and education of the household head. Each of the latter variables was positively associated with market sourcing of protein-rich foods. Reliance on own production had a negative association with urban location, age, and health of household heads, improved sanitation, fish money but was positively associated with reliance on employment income and Covid cases.

The patterns for iron-rich food were rather different. As for other nutrients, urban living, improved sanitation, household size, and positive income changes were positively associated with higher iron intake and reliance upon markets. However, there were no significant associations with income sources, household head gender, age or education, Covid cases, or Covid-induced hoarding. We found a negative relationship between Covid policy stringency and iron-rich food intake but this seems to be driven through own production, not market sourcing. This particular effect is also suggested by the positive association between Covid cases and own production and a negative association with market sourcing. Reliance on own production is also positively associated with a deterioration in the health of a household resident.

The positive relationship between the Ramadan dummy and FCS, iron, and Vitamin A and the negative association with HFIAS can be attributed to households trying to ensure they maintained a diversified diet during a period of fasting but this did not seem to include protein.

## Discussion

The primary strengths of this study are its longitudinal nature and its analysis of food indices disaggregated by source within a particular social group. This enables our results to identify specific dimensions of inequality (such as household resources) that limit the resilience of households in the face of the pandemic shock. Our findings, therefore, corroborate many of those from the literature and provide some contextual nuances.

We found that functioning food markets and adoption of a range of production and consumption coping mechanisms were paramount (13). As only 9% of households in our survey received government aid, we were unable to rigourously test previous findings on the importance of social safety nets in the early stages of the pandemic (9). We simply note the small number of recipients and that all of these also received remittances. To the extent that pandemic restrictions impair markets or coping strategies, they may limit dietary diversity (19), especially in rural areas (21). This may be through more restricted food choices or higher costs of transport in rural areas to find work.

Although we found some suggestions of gender-specific associations with coping mechanisms and Vitamin A consumption, we could not replicate findings elsewhere in the literature (20) that gender was more widely associated with vulnerability, as our dataset had only a small number of sole female household heads (Table 4). However, we did confirm the continued importance of agriculture as an income source and food source (Table 6) (25–27). The maintenance of dietary diversity through seasonal switching between own and market sources is well-documented (24), but our results highlight which particular households and for which particular food groups (Table 9).

Our descriptive results also suggest a range of household, spatial and temporal factors that drive dietary behavior over time. All households were from the Mandinka social group and frequently featured large, mobile and polygamous households usually led by male household heads with a low level of education and a poor state of health (Table 4). Diets were heavily dependent upon staples across all locations and market food sources, especially in urban areas (Tables 5, 6). As Kundu et al. (21) found in Bangladesh, we found that Gambian rural micronutrient intake was consistently lower than in urban households (Table 7). Whilst this echoes findings of higher food insecurity in rural areas of The Gambia (43), growing inter-household dispersion from May to July was not location specific (Table 7).

Our findings did not suggest the same degree of food insecurity in terms of availability identified in national Gambian surveys conducted earlier in the pandemic (6, 42). Food security in our survey was manifest in terms of restricted food choice, rather than quantity (Tables 5, 7), but households employed coping strategies to ensure stable nutritional intake during Ramadan. Income falls occurred mainly in February and September and were concentrated in a sizeable minority of the survey population. Rural households were more reliant upon employment and production income and susceptible to multiple monthly declines, especially after June. Less than 50% of households received remittances more than once and less than 9% received Government aid (Table 8). The wide dispersion of disposable spending money suggests that some household budgets were more squeezed by rising food prices than others. This may mirror Dou et al.'s (14) finding of varying resilience capacity within income cohorts, not just across them though we were not able to replicate Crush and Si's (22) finding that this dispersion may reflect pre-existing food insecurity driven by age, gender, and occupation.

This array of factors highlights the multi-dimensional pandemic impact pathways (20) and had a strong bearing on our regression results. We identified significant associations between dietary behavior and six key variables: location, improved sanitation, household size, changes in monthly income, Covid policy stringency, and Covid cases (Table 9). These associations are suggestive of spatial, temporal, and pandemic effects but the magnitude of these varied by dietary metric and the specific Covid impact. These findings echo those of Teachout and Zipfel (19), which suggest that lockdown restrictions may have exacerbated food security issues for some households and those of Egger et al. (20) which suggest these effects persisted long after policy easing. We found that food insecurity was manifest in terms of restricted choices (14), rather than availability or affordability (13).

In terms of psychological effects, our HFIAS results (Table 7) suggested that tighter policy stringency and fear of pandemic-induced hoarding encouraged wider use of dietary coping mechanisms to maintain food choices. As Covid cases did not rise until toward the end of the study, they had less effect on most dependent variables but were associated with greater reliance on own production for some food types. This echoes the findings of Madzorera et al. (27). The positive association between the incidence of household illness and coping strategies and reliance on own produced iron-rich foods may suggest that behavioral change actually occurs only when a health risk is perceived to be personal.

Policy stringency had a negative association with most dietary diversity scores and with reliance on own production of all food types as found elsewhere in SSA (27), except those rich in protein where market sources were negatively associated (Table 9). This appears to confirm Teachout and Zipfel's (19) finding that policy restrictions may undermine long-term resilience. Increased national Covid cases were associated with own production of protein and iron-rich food scores and perceived fears of market hoarding Covid was also positively associated with own production of Vitamin A-rich food scores. These differences may not only reflect seasonality to some extent but also suggest that the impact of each different aspect of Covid needs to be assessed by a specific food group.

Income changes were also significantly associated with several dependent variables (Table 9) but it is unclear if these can be attributed directly to the pandemic. Although our variables may not explain changes in the intake of nutrients, they may indicate changes in food sourcing. An important finding is that the associations were not consistent by nutrient type or location. Urban location was associated with improved dietary outcomes using all metrics (21), except Vitamin A-rich foods, which were largely sourced from own production. Iron-rich food intake was not associated with income source or Covid cases but reliance on own production sources for iron was positively associated with household sickness and Covid cases. Although we found that female-headed households were more likely to eat Vitamin A-rich foods and adopt more coping strategies, the small number of observations does not allow us to definitively determine a gender effect (22).

As well as spatial factors, the ability to adapt dietary and sourcing habits appears to be subject to household-specific constraints (14). Households with improved services (which may be deemed a wealth proxy) and larger households (which may be deemed an income-earning capacity proxy) were more resilient in terms of diversity and nutritional outcomes, though most households took steps to ensure stable food intake during Ramadan (Table 9). Although we found no relationship between migration and dietary outcomes *per se*, it is possible that migration had an indirect income effect through remittances.

Our results suggest that Covid policy restrictions and the rise in cases have had a negative effect on dietary outcomes and altered food sourcing behavior to some degree (19, 20). However, neither Covid policy nor cases appear to have affected the frequency of market visits (especially in rural areas) or of eating out (Tables 6, 9). In the face of the pandemic, households have adopted a range of food-specific coping strategies (Tables 6, 7), including dynamically switching between sources as available (13). Therefore, the effects of the pandemic have been filtered through location and household-specific wealth and income proxies that have constrained household resilience.

In terms of policy implications, our findings reiterate those from elsewhere in SSA (19) that there is scope for more sophisticated targeted of Government aid as it does not appear to have reached most households and certainly not those most in need, particularly those with higher dependence on employment income and no access to remittances. We do not try to suggest that the absence of social safety nets was a cause of dietary hardship (due to insufficient data) simply that not many received any aid and those who did had alternate safety nets. The fact that there is some inconsistency across food groups may also suggest that a strategy to address one dimension of micronutrient deficiency is not necessarily one that can address others.

We acknowledge a number of limitations in our study. Our findings are limited by the size of our sample population, reliance on those with regular access to a mobile phone, the use of convenience and snowballing techniques, and the small number of female household heads, as well as the fact that Covid incidence was relatively low and local policy relaxed for most of the survey period. Nevertheless, circumstances did make collection and co-ordination rather problematic. We had hoped to follow-up on some of the questions raised in our survey through individual interviews and focus groups but resources, timing, and Covid restrictions precluded this. We also recognize that we relied on nutritional frequency proxies in terms of dietary intake at the household level, rather than measuring individual intake or nutritional outcomes directly. We have tried to take reasonable steps to address issues in our dataset but recognize that as not all potential causes of error can be fully mitigated (54), these may limit the robustness of our results.

## Conclusions

This longitudinal study examines the multi-dimensional impact pathways of the Covid pandemic within one ethnic group located in urban and rural areas of The Gambia. It thereby contributes to the literature in terms of improved understanding of the interaction between food environments, lockdown policy regimes, and household coping strategies in specific contexts (20).

Food insecurity was manifest during the 9-month survey period mainly in terms of lack of choice and nutritional variety, rather than quantity. Our regression analysis demonstrates that dispersion of household dietary outcomes and sourcing strategies were associated with location, improved sanitation, household size, changes in monthly income, Covid policy stringency, and Covid cases. An important finding is that there were variations in food group consumption by location and by food nutrient group. Rural communities were more likely to eat more healthy millets (sourced from own production) but much less likely to consume dairy products or roots and tubers. Access to own production was important for Vitamin A-rich foods but higher incomes and markets were key for protein and hem-iron-rich foods. Tighter policy stringency was negatively associated with dietary diversity and positively associated with increased reliance on a range of coping mechanisms. Resilience was higher in larger households and those with access to improved water and sanitation. Higher consumption of protein-rich foods and greater reliance on own produced iron-rich foods was associated with the number of Covid infections.

As well as reaffirming findings from other contexts, this paper highlights how different aspects of the pandemic affect dietary diversity in different ways and that impact pathways are contingent upon an array of spatial and household-specific variables. Through further research, these findings can hopefully serve as a platform through which targeted policy measures can be designed to address food-specific deficiencies and the inequalities in resilience capacity that has been so widely exposed by the pandemic.

## Data availability statement

The raw data supporting the conclusions of this article will be made available by the authors, without undue reservation.

## Ethics statement

All procedures including the use of recorded audio consent and social distancing protocols were approved by the Scientific and Ethical committees of MRC Gambia / London School of Hygiene and Tropical Medicine, the Gambia Government/MRC Joint Ethics Committee, and by the Department of Land Economy at the University of Cambridge.

## Author contributions

All authors listed have made a substantial, direct, and intellectual contribution to the work and approved it for publication.

## Funding

This project was funded by the Biotechnology and Biological Sciences Research Council (BBSRC)-Global Challenges Research (GCRF), Research on Millets and Nutritional Enhancement Traits for Iron bioavailability (MillNET_i) (April, 2019 – October, 2021) Grant Reference BB/S013954/1 and Transforming India's Green Revolution by Research and Empowerment for Sustainable food Supplies, funded by the Global Challenges Research Fund (TIGR2ESS) (October, 2021 - March, 2022) Grant reference BB/P027970/1. The funding institutions had no part in the design of the survey or submission of this paper.

## Conflict of interest

The authors declare that the research was conducted in the absence of any commercial or financial relationships that could be construed as a potential conflict of interest.

## Publisher's note

All claims expressed in this article are solely those of the authors and do not necessarily represent those of their affiliated organizations, or those of the publisher, the editors and the reviewers. Any product that may be evaluated in this article, or claim that may be made by its manufacturer, is not guaranteed or endorsed by the publisher.

## Footnotes

^1^MillNET_i was funded by the Biotechnology and Biological Sciences Research Council (BBSRC)-Global Challenges Research (GCRF) from April, 2019 to October, 2021 (Grant Reference BB/S013954/1). Additional funding was received October, 2021 − March, 2022 from the Transforming India's Green Revolution by Research and Empowerment for Sustainable food Supplies research project (TIGR2ESS) also funded by the Global Challenges Research Fund (Grant reference BB/P027970/1).

^2^Measured from 0 to 100, this scale of pandemic measures includes school and work closures, public event and travel bans, information, testing and vaccination campaigns (41).

^3^We are aware that, as a consequence, we do not address intra-household inequities.

^4^Kiang West Women's Migration Study (WMS) ((SCC1389v2) and a Feasibility study (SCC1222v2 L2013.56)) conducted by Dr Sarah Dalzell at the MRC Gambia unit.

^5^There were no refusals.

^6^Household composition data was collected on the first call and updated for any changes in health or employment status and household entrants or exits on each subsequent visit. In accordance with WFP guidelines (48), consumption data was collected on the basis of the previous 7 days and all other data was based on the previous month.

^7^These were preferred to the Household Dietary Diversity (HDDS) score as gauge of nutrition (50).

^8^We had insufficient observations to use government aid.

^9^Other responses had insufficient variability (e.g., prices) or overlapped other variables (e.g., jobs).

^10^Three male heads were absent during the survey but were consented prior to consenting their wives.

^11^During subsequent calls, we found 84 instances of a new health condition – 18 percent of which related to hypertension, diabetes, respiratory illness or obesity.

^12^As we were wary of difficulties in estimating numbers in a remote survey and our pilot surveys indicated some reluctance, households were not asked to quantify income, only to cite top three sources.

^13^Protein rich food sourced from own production and iron rich food sourced from markets respectively.
